# Shifting Inequalities? Parents’ Sleep, Anxiety, and Calm during the COVID-19 Pandemic in Australia and the United States

**DOI:** 10.1177/1097184X21990737

**Published:** 2021-02-02

**Authors:** Leah Ruppanner, Xiao Tan, William Scarborough, Liana Christin Landivar, Caitlyn Collins

**Affiliations:** 1 University of Melbourne, Parkville, VIC, Australia; 2 University of North Texas, Denton, TX, USA; 3 Maryland Population Research Center, College Park, MD, USA; 4 Washington University in Saint Louis, St. Louis, MO, USA

As a cultural ideal, hegemonic masculinity positions men as breadwinners in the gender order—a position that systematically benefits men and disadvantages women. Because economic success is key to performing masculinity (Connell 2005), the COVID-19 pandemic and its economic fallout offer an opportunity to evaluate shifting gender dynamics amidst rapid changes in employment and domestic demands for heterosexual couples with children. Closures of schools, daycare facilities, and workplaces around the world shifted more paid and unpaid work into the home, leading journalists and academics to question whether the pandemic would be a catalyst to “un-stall” the gender revolution. Specifically, they wondered if men would take on more domestic work, generating a more equal gender division of household labor (Smith and Johnson 2020). In this essay, we argue that traditional gender roles were reinforced for U.S. parents but were eroded for Australian parents—with disparate consequences for their well-being during the first few months of the pandemic.

Emerging research shows that fathers in heterosexual couples did increase their contribution to domestic labor during the pandemic, though not as much as mothers, who shouldered the greater share of COVID-generated childcare, housework, and homeschooling (Alon et al. 2020; Carlson, Petts, and Pepin 2020; Craig and Churchill 2020; Heggeness and Fields 2020). This gender gap in pandemic care work corresponded to a dramatic reduction in women’s paid labor hours, particularly among mothers of young children (Collins et al., forthcoming), and to higher labor force exit rates of mothers compared to fathers (Landivar et al. 2020). Rather than spurring greater equality, COVID-19 appears to reinforce traditional expectations of men as financial providers and women as homemakers.

We make two contributions to this body of research. First, we examine whether and how gendered trends in time use and economic precarity for mothers and fathers during the pandemic manifest in disparate health outcomes (Springer, Stellman, and Jordan-Young 2011) measured through anxiety, calmness, and sleep quality. Job loss and increased domestic labor for men under COVID-19 may threaten traditional norms of masculinity that value fathers as economic providers but not caregivers. Reinforcing hegemonic masculinity, only job loss, and not changes in domestic loads, may increase men’s anxiety and sleep, and reduce their sense of calm. Alternatively, COVID-19 may restructure gender norms such that fathers assume more housework and childcare, negatively affecting their anxiety, sleep, and calmness. Finally, the COVID-19 pandemic may equalize gender roles, making mothers’ and fathers’ health experiences more alike in this time of crisis. Of these possibilities, our analysis suggests that U.S. parents’ time spent in paid work, housework, and childcare served to reinforce hegemonic masculinity, while Australian parents reshuffled gender roles in these domains. In each case, this had repercussions for fathers’ and mothers’ well-being.

Second, we adopt a cross-national, comparative approach to evaluate parents’ experiences of anxiety, sense of calm, and disrupted sleep in Australia and the United States—two “gender regimes” (Connell 2002) rooted in norms of gender traditionalism and hegemonic masculinity (Connell 1998; Scarborough, Sin, and Risman 2019)—to shed light on the embodied emotional toll of COVID-19. Australia and the United States are both liberal welfare regimes with dual-earner work cultures and women-caregiver parenting cultures that reinforce hegemonic masculinity. By May of 2020, government leaders in both countries had initiated varying degrees of lockdown to mitigate the spread of the infection, including school and workplace closures. Beyond this, however, the countries diverged. The United States exhibited strong political polarization, a decentralized approach, and a patchwork of state measures to control infection rates (Bariola and Collins, forthcoming). This stands in stark contrast to Australia’s unified federal and state messaging and policy implementation. Except for the state of Victoria, which experienced a second wave of infections and subsequent strict lockdowns, transmission of the virus has been minimal in Australia (Figure 1). Meanwhile, infection rates in the United States are the highest in the world—and still growing.

**Figure 1.**
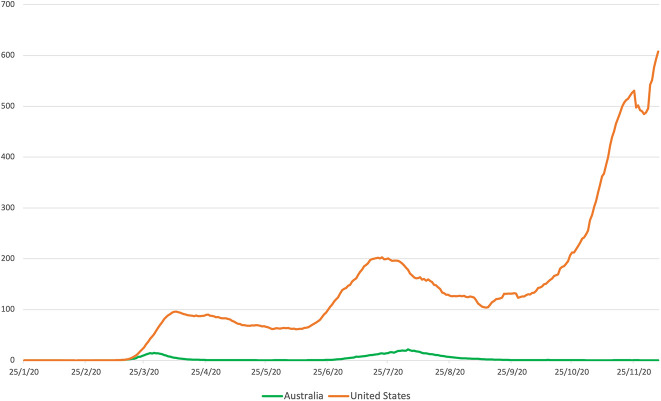
Daily new confirmed COVID-19 cases per million people, Australia and the United States. Source: COVID-19 Data Repository by the Center for Systems Science and Engineering at John Hopkins University (2020).

Although the two nations had divergent infection rates, both countries experienced a sharp economic downturn and huge unemployment spikes. Women account for a larger share of employment losses due, in part, to occupational segregation of women into hard-hit industries, including tourism, service, and retail (United Nations 2020)—generating a “shecession” in both nations (Gupta 2020). Given these similarities, both countries were positioned to revert to traditional breadwinner norms at the expense of mothers’ emotional well-being in response to the novel coronavirus. We find, however, that only in Australia did fathers’ larger uptake of domestic labor hold steady over time and contribute to Australian fathers’ elevated anxiety and worse sleep—patterns similar to those of American mothers. U.S. fathers’ anxiety and sleep were only influenced by job loss, reinforcing norms of hegemonic masculinity.

## Gendered Consequences of the Pandemic on Job Change and Family Labor

We sampled parents from the YouGov (a global leader in survey research) panels in Australia and the United States in May and September 2020. The samples were drawn against key demographics in both countries to ensure national representativeness. The timeframe captures (a) the height of the first lockdown and (b) four months later, when schools reopened for the new academic year in the United States (in person, virtually, or a mix of the two; Collins et al. 2021) while Victoria, Australia was under a strict second lockdown. For this study, we limited the analytical sample to only those parents with childcare responsibilities (N=764 for Australia; N=611 for the U.S.).^1^

**Figure 2.**
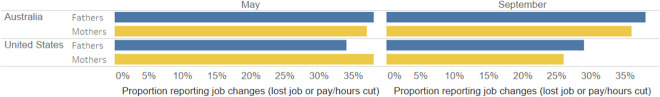
Job changes due to COVID-19. Source: Primary data collection from YouGov: Australian and U.S. panels (2020).

As expected, parents in our study experienced significant changes in their job, housework, and childcare activities. In May, around 35% of the surveyed parents in both countries reported losing pay, work hours, or employment. These patterns were similar for fathers and mothers (Figure 2). For our sample, then, fathers and mothers were similarly vulnerable to employment disruption during the first lockdown. In September, both fathers and mothers in the United States reported a decrease in job instability, reflecting job recovery since May. The proportion of parents reporting job instability and pay or hours reductions in Australia remained steady between May and September.

**Figure 3.**
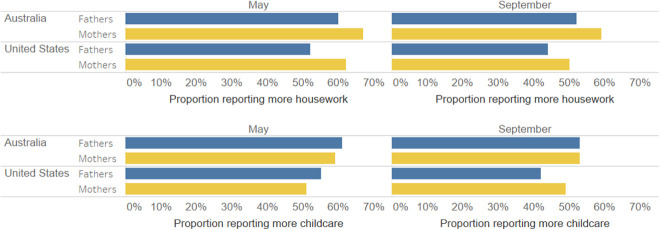
Housework and childcare changes due to COVID-19. Source: Primary data collection from YouGov: Australian and U.S. panels (2020).

In May, in both Australia and the U.S. fathers report picking up less housework but more childcare than did mothers (Figure 3). In September in Australia, fathers still report doing less housework than mothers report doing, but fathers and mothers reported a relatively equal level of childcare. In contrast, in the United States, fathers reported smaller contributions in both dimensions. From May to September, American fathers appear to have retreated from childcare, perhaps to dedicate more energy to resuming paid work (Figure 2). This shift suggests a return to traditional childcare arrangements.

## Job Change, Increased Family Labor, and Parents’ Health During the Pandemic

We applied multilevel (varying intercept) ordered logistic regression models to test the relationship between job change, increased housework, and increased childcare loads on fathers’ and mothers’ anxiety, restless sleep, and calmness. We report substantive results in Table 1 (see our additional Appendix files online: Table S1 for descriptions of our variables and Table S2 and Table S3 for full results).^l^

**Table 1.** Multilevel Ordered Logistic Regression Results.

	Anxiety			Restless Sleep			Calmness		
	Fathers	Mothers	Sig. Gender Difference	Fathers	Mothers	Sig. Gender Difference	Fathers	Mothers	Sig. Gender Difference
Australia (fathers=383; mothers=381)									
Job lost or pay/hours cut	0.56*	0.24		0.61*	0.27		-0.83***	0.54	***
Increase in housework	0.96***	-0.06		1.06***	0.32	*	0.26	-0.31	
Increase in childcare	0.82***	0.73**	**	0.31	0.82**		-0.31	0.07	
United States (fathers=289; mothers=322)									
Job lost or pay/hours cut	0.86**	1.15***		0.66**	1.11***		-0.37	-0.46	
Increase in housework	0.10	0.75**		0.06	0.74**		0.00	-0.41	
Increase in childcare	0.73	0.46		0.19	0.05		-0.05	-0.16	

*Note*. *p<.1, **p<.05, ***p<.01. Source: Primary data collection from YouGov Australian and American panels (2020). Only the substantive results are shown in this table. Controls include age, marital status, education, income and a wave dummy. The full results can be found in the Table S2 and Table S3 (online appendix).

Our findings suggest that the pandemic took different tolls on mothers and fathers in Australia and the United States. In Australia, both economic challenges (job changes) and growing domestic duties (housework and childcare) related to worse health outcomes for *both* mothers and fathers. During this period, Australian fathers’ growing housework and childcare responsibilities have a stronger relationship to anxiety and restless sleep for them than for mothers, while job loss reduces fathers’ calmness more than it does for mothers. These patterns are best understood through traditional breadwinner/homemaker norms. Changes across work and domestic life under COVID-19 less severely affected mothers, who have long navigated employment disruptions and larger housework and childcare demands. By comparison, Australian fathers negotiating job loss *and* larger domestic loads, perhaps for the first time, experienced worse mental health.

In contrast to Australia, we observe gendered patterns of pandemic mental health consequences in the United States that suggest the continuation of traditional norms surrounding masculinity—even in the face of massive economic and social changes. Growing housework or childcare burdens did not affect American fathers’ mental health, while growing housework demands predicted higher incidence of anxiety and worse sleep for U.S. mothers (note: no association for calmness). For U.S. fathers, only job changes predicted worse mental health outcomes, a relationship also significant for U.S. mothers. Collectively, our results from U.S. parents indicate that fathers’ health outcomes remain tied to their careers, reflecting the endurance of conventional norms of hegemonic masculinity rooted in fathers’ breadwinning during this period of unparalleled economic and social upheaval. U.S. mothers’ health outcomes are tied to both work and family outcomes, resonating with intensive mothering norms that convey ideal motherhood as comprising exhaustive caregiving alongside career aspirations (Hays 1996).

## Looking Forward: COVID-19 and Fathers’ and Mothers’ Health

We provide preliminary evidence that COVID-19 eroded traditional gender roles in Australia but reinforced them in the United States. The pandemic has been particularly damaging to U.S. mothers’ and Australian fathers’ anxiety and sleep. Since the time of the survey, U.S. mothers have continued to shoulder enormous demands with school closures, limited childcare, and lofty employer expectations. Our results suggest these competing demands will continue to compound anxiety and sleep problems, which are dire indicators for U.S. mothers and their health. Given the re-opening of the Australian economy, these added burdens may abate—but Australian fathers’ greater childcare and housework contributions may persist. The COVID-19 pandemic has been far from a catalyst for unequal gender regimes to reinvent gender roles. Our research shows, however, that Australia may have shifted toward more egalitarian gender relations while the United States remains tethered to hegemonic gender ideals, perpetuating existing systems of gendered power and inequality.

## Supplemental Material

Supplemental Material, sj-docx-1-jmm-10.1177_1097184X21990737 - Shifting Inequalities? Parents’ Sleep, Anxiety, and Calm during the COVID-19 Pandemic in Australia and the United States Click here for additional data file. Supplemental Material, sj-docx-1-jmm-10.1177_1097184X21990737 for Shifting Inequalities? Parents’ Sleep, Anxiety, and Calm during the COVID-19 Pandemic in Australia and the United States by Leah Ruppanner, Xiao Tan, William Scarborough, Liana Christin Landivar and Caitlyn Collins in Men and Masculinities

## Note

1. A portion of these respondents were sampled at both time points, but we present the results for our entire sample because the substantive conclusions are largely equivalent. All trends are presented separately for each time point to avoid issues related to serial correlation between panel respondents.
