# Supplementary material for: Shifting Inequalities? Parents’ Sleep, Anxiety, and Calm during the COVID-19 Pandemic in Australia and the United States
Source: Men Masc. 2021 Feb 2:1097184X21990737. doi: 10.1177/1097184X21990737 (PMC7862912; doi:10.1177/1097184X21990737)
Supplement: Supplemental Material, sj-docx-1-jmm-10.1177_1097184X21990737 - Shifting Inequalities? Parents’ Sleep, Anxiety, and Calm during the COVID-19 Pandemic in Australia and the United States [file sj-docx-1-jmm-10.1177_1097184X21990737.docx]

**APPENDIX**

**Table S1. Full list of variables**

|  | Supplementary information | Codes |
| --- | --- | --- |
| *Dependent variables* | | |
| Anxiety | Respondents were asked to indicate the amount of time they felt anxious in the past week. | 1=None or almost none of the time;  2=Some of the time;  3=Most of the time;  4=All or almost all of the time |
| Restless sleep | Respondents were asked to indicate the amount of time they had restless sleep in the past week. | 1=None or almost none of the time;  2=Some of the time;  3=Most of the time;  4=All or almost all of the time |
| Calmness | Respondents were asked to indicate the amount of time they felt calm in the past week. | 1=None or almost none of the time;  2=Some of the time;  3=Most of the time;  4=All or almost all of the time |
| *Key Independent variables* | | |
| Job lost or pay/hours cut | Respondents were asked whether they lost job or had pay or hours reduced due to COVID-19 lockdown. | 0=No;  1=Yes |
| Increase in housework | Respondents were asked about the change in housework since social distancing measures began (1=doing much less to 5=doing much more). The responses were recoded into a dummy variable, with 1 representing doing more or much more housework. | 0=No;  1=Yes |
| Increase in childcare | Respondents were asked about the change in childcare since social distancing measures began (1=doing much less to 5=doing much more). The responses were recoded into a dummy variable, with 1 representing doing more or much more childcare. | 0=No;  1=Yes |

| *Controls* | | |
| --- | --- | --- |
| Age | We measured age using a series of dummy variables: 25 to 34, 35 to 44 (the reference group), 45 to 64. | 0=No;  1=Yes |
| Marital status | We measured marital status through a dummy variable (1=married). | 0=Not married;  1=Married |
| Education | Respondents were asked about their educational qualifications, but the choices were different given the different education systems in the two countries. We regrouped these responses into one single dummy variable, with the value 1 assigning to those who hold a university degree or above. | 0=Below university;  1=University or above |
| Household income | We grouped the responses into four categories: low income (less than AU$50k or US$40k), middle income (between AU$50k and AU$100k, or between US$40k and US$80k; the reference group), high income (more than AU$100k or US$80k), and missing income. The dummy variable of missing income was created to include those people who chose to not report their household income. We used this dummy variable to avoid losing observations. | 0=No;  1=Yes |
| Time dummy |  | 0=May;  1=September |

**Table S2. Multilevel ordered logistic regression results for Australia (fathers=383; mothers=381)**

|  | Anxiety | | | Restless Sleep | | | Calmness | | |
| --- | --- | --- | --- | --- | --- | --- | --- | --- | --- |
|  | Fathers | Mothers | Sig. gender difference | Fathers | Mothers | Sig. gender difference | Fathers | Mothers | Sig. gender difference |
| Job lost or pay/hours cut | 0.56* | 0.24 |  | 0.61* | 0.27 |  | -0.83*** | 0.54 | *** |
| Increase in housework | 0.96*** | -0.06 |  | 1.06*** | 0.32 | * | 0.26 | -0.31 |  |
| Increase in childcare | 0.82*** | 0.73** | ** | 0.31 | 0.82** |  | -0.31 | 0.07 |  |
| Age: Between 18 and 29 | 0.39 | 0.53 |  | -0.88 | -0.32 |  | 0.12 | 0.26 |  |
| Age: Between 45 and 64 | -0.65* | 0.01 |  | -0.02 | -0.02 |  | -0.20 | 0.22 |  |
| Married | 0.10 | -0.54 |  | -0.62 | -1.27** |  | 0.16 | 0.31 |  |
| University or above | 0.15 | 0.40 |  | -0.45 | 0.15 |  | 1.16*** | 0.15 |  |
| Income: Less than AU$50k or US$40k | -0.47 | -0.69* |  | -0.28 | -1.25** |  | 0.72* | 0.25 |  |
| Income: More than AU$100k or US$80k | -0.71** | -1.02** |  | -0.42 | -0.79 |  | -0.05 | 0.07 |  |
| Missing income | -0.42 | -0.06 |  | -0.28 | -0.49 |  | -0.51 | -0.31 |  |
| September wave | 0.50** | 0.57** |  | 0.02 | 0.91*** | ** | -0.58** | 0.01 | * |

*Note*. *p<.1, **p<.05, •••p<.01. Source: Primary data collection from YouGov Australian and American panel (2020).

**Table S3. Multilevel ordered logistic regression results for the United States (fathers=289; mothers=322)**

|  | Anxiety | | | Restless Sleep | | | Calmness | | |
| --- | --- | --- | --- | --- | --- | --- | --- | --- | --- |
|  | Fathers | Mothers | Sig. gender difference | Fathers | Mothers | Sig. gender difference | Fathers | Mothers | Sig. gender difference |
| Job lost or pay/hours cut | 0.86** | 1.15*** |  | 0.66** | 1.11*** |  | -0.37 | -0.46 |  |
| Increase in housework | 0.10 | 0.75** |  | 0.06 | 0.74** |  | 0.00 | -0.41 |  |
| Increase in childcare | 0.73 | 0.46 |  | 0.19 | 0.05 |  | -0.05 | -0.16 |  |
| Age: Between 18 and 29 | 0.15 | 0.04 |  | 0.15 | 0.39 |  | -0.07 | 0.30 |  |
| Age: Between 45 and 64 | -1.23** | -1.27** |  | -0.94** | -0.22 |  | 0.20 | 0.74** |  |
| Married | -0.04 | 0.79* |  | 0.03 | 0.03 |  | -0.14 | 0.59* |  |
| University or above | 0.62 | -0.03 |  | 0.28 | 0.04 |  | -0.53 | 0.01 |  |
| Income: Less than AU$50k or US$40k | 1.44*** | 0.62 |  | 0.75* | 0.50 |  | -0.13 | 0.47 |  |
| Income: More than AU$100k or US$80k | 0.04 | 0.75 |  | -0.26 | -0.63 |  | -0.01 | 0.62 |  |
| Missing income | 0.48 | 0.46 |  | 0.91 | -0.30 |  | -0.75 | 0.04 |  |
| September wave | 0.83** | 0.50* |  | 0.21 | -0.07 |  | -0.67** | -0.15 |  |

*Note*. *p<.1, **p<.05, •••p<.01. Source: Primary data collection from YouGov Australian and American Panels (2020).
